# Are Sputum High Mobility Group Box 1 and D-Dimer Changes Relevant Markers of Tissue Damage and Fibrinolysis in Cystic Fibrosis?

**DOI:** 10.3390/pathophysiology33030053

**Published:** 2026-07-21

**Authors:** Sante Di Gioia, Annalucia Carbone, Pamela Vitullo, Domenico Tierno, Domenico Larobina, Gabriele Grassi, Mario Grassi, Massimo Conese

**Affiliations:** 1 Department of Clinical and Experimental Medicine, University of Foggia, I-71122 Foggia, Italy; sante.digioia@unifg.it (S.D.G.); annalucia.carbone@unifg.it (A.C.); 2 Cystic Fibrosis Support Center, Ospedale “G. Tatarella”, I-71042 Cerignola, Italy; pamelavitullo@gmail.com; 3 Department of Medicine, Surgery and Health Sciences, University of Trieste, Strada di Fiume 447, I-34149 Trieste, Italy; domenico.tierno@units.it (D.T.); ggrassi@units.it (G.G.); 4 Institute of Polymers, Composites and Biomaterials, National Research Council of Italy, P.le E. Fermi 1, I-80055 Portici, Italy; domenico.larobina@cnr.it; 5 Department of Engineering and Architecture, University of Trieste, Via Valerio 6/A, I-34127 Trieste, Italy; mario.grassi@dia.units.it

**Keywords:** cystic fibrosis, HMGB1, D-dimer, biomarker, damage, inflammation, fibrinolysis, lung disease

## Abstract

Background/Objectives: Cystic fibrosis (CF) is a genetic disease whose hallmarks include chronic inflammation of the airways causing tissue damage with increased levels of alarmins in the respiratory secretions, as well as activation of the coagulation/fibrinolytic systems that are linked to inflammation. However, no information is available about the fibrinolytic system and how fibrinolysis is involved in airway injury. On the road to understanding this issue, we determined levels of two key markers, High Mobility Group Box 1 (HMGB1), an alarmin, and D-dimers, associated with fibrin breakdown and airway inflammation, in the sputum from patients with cystic fibrosis. Methods: Sputum samples were collected from 13 individuals with CF and subjected to two different treatment protocols. In the first protocol, the sample was treated with dithiothreitol (DTT) and then centrifuged in order to collect the supernatant (SED). In the second protocol, the sample was centrifuged and the supernatant was obtained (SE). The pellet obtained was treated with DTT and then centrifuged in order to collect the supernatant (SPE). ELISA assays were performed on all samples. Results: HMGB1 and D-dimer levels were significantly lower in supernatants from sputum centrifuged before DTT treatment (SE) compared to those processed after DTT (SPE and SED). However, no significant difference was observed between SPE and SED samples for both markers. D-dimer levels in SED correlated positively with FEV_1_ and blood monocyte counts. Conclusions: Direct sputum processing with DTT may be a useful procedure for assessing biomarkers of inflammation and fibrinolysis in CF.

## 1. Introduction

Cystic fibrosis (CF) is a genetic disease with autosomal recessive heritability which affects many organs and tissues with absorptive/secretive properties, including the pancreas, lungs, sweat glands, and the digestive and reproductive tracts [1]. Nevertheless, lung disease is the main determinant of morbidity and mortality of CF patients. CF lung disease causes thick, sticky mucus buildup, leading to chronic coughing (often with sputum), frequent lung infections and shortness of breath [2]. Constant inflammation of the airways, leading to damage over time, is the pathological hallmark of this disease [3]. Proteases and oxidants produced by airway epithelial cells and resident as well recruited immune cells provoke continuous damage to airway epithelia and underlying structures, leading to high levels of mediators in the respiratory secretions [1,4]. In particular, besides inflammatory cytokines and chemokines, CF airway secretions are replete with alarmins, i.e., danger-associated molecular patterns liberated or secreted by damaged cells [5,6]. One of the most important alarmins is High Mobility Group Box 1 (HMGB1), which is located in the nucleus in homeostatic conditions, but it is freed from damaged cells or even secreted [7]. Macrophages and neutrophils are the cell types involved in HMGB1 production. HMGB1 is present in CF sputum and bronchoalveolar lavage fluid (BALF) obtained from CF airways [5,8,9], and its levels correlate with progression of lung function decline [10].

Blood coagulation is known to be elicited by inflammatory processes and is involved in the production of inflammatory mediators itself, thus perpetuating a vicious inflammatory cycle. Fibrinolysis follows coagulation in order to break down fibrin clots, thus exerting a pro-resolution or an anti-inflammatory function. However, the components involved in this process, particularly the enzyme plasmin, can initiate pro-inflammatory responses, including cytokine release and cell migration [11,12].

Biomarkers of inflammation, danger, and tissue repair can be studied in respiratory secretions, such as sputum and BALF [13,14]. These matrices are the most useful ones to describe pathophysiology of CF lung disease at the local level, but might also be important for diagnostic and prognostic purposes, as well as to gauge therapeutic interventions. While BALF collects secretions from the more distal airways, sputum is representative of the upper airways (bronchi/bronchioles), where CF lung disease occurs. However, sputum is contaminated by saliva and has to be dissolved to free biomarkers from its complex matrix. The treatment of sputum samples with reducing agents such as dithiothreitol (DTT) so as to obtain a chemical homogenization before analysis may affect the validity of biomarker measurements, in particular tumor necrosis factor (TNF)-α, leukotriene B4 (LTB4), and myeloperoxidase (MPO) [15]. Therefore, before considering HMGB1 and D-dimers as potential biomarkers of CF lung disease, it is essential to determine whether the commonly used reducing agent DTT alters their measurable concentrations. Assessing the analytical impact of sputum processing is thus a necessary methodological step to ensure the reliability and interpretability of biomarker studies in this matrix [15].

Accordingly, the aims of the present study were twofold: first, to investigate HMGB1 and D-dimers in CF sputum as markers of tissue damage and fibrinolysis, respectively; and second, to determine whether DTT, a reducing mucolytic agent routinely used for sputum homogenization, affects their detection and quantification. Notably, although the influence of DTT on several sputum biomarkers has been described, the measurement of D-dimers in CF sputum after treatment with reducing agents has not been previously evaluated.

## 2. Materials and Methods

### 2.1. Patients and Methods

CF patients (>18 years) were recruited at the Cystic Fibrosis Support Center of the “G. Tatarella” Hospital (Cerignola, Italy). CF patients with *F508del*/*F508del* and *F508del*/other mutation genotypes were included in this study. Enrolled patients had a confirmed CF diagnosis based on sweat chloride testing and/or genotyping, and were on CFTR modulator therapy (Trikafta) for at least 15 days prior to sample acquisition. The study was approved by the Ethics Committee of the Riuniti Hospital of Foggia (no. 124/CE/2023, issued on 26 July 2023) and was conducted in compliance with the current Good Clinical Practice standards and in accordance with the relevant guidelines and regulations and the principles set forth under the Declaration of Helsinki (1989). During this study, the privacy rights of participants were observed and informed consent was obtained.

Spontaneous sputum samples were obtained and processed. Sputum samples were subjected to two different treatment protocols. In the first protocol (*n* = 5–6), after mechanical removal of saliva, the sample was treated with DTT and then centrifuged in order to collect the supernatant (hereinafter referred to as SED). In the second protocol (*n* = 7–8), after removal of saliva, the sample was centrifuged and the supernatant was obtained (hereinafter referred to as SE). The pellet obtained was treated with DTT and then centrifuged in order to collect the supernatant (hereinafter referred to as SPE). The liquefaction with DTT was performed as previously described [16]. Samples intended for treatment with the mucolytic were diluted with 2.5 volumes of sterile saline. An equal volume of 0.1% DTT solution was then added to this mixture. The 0.1% DTT solution was obtained by diluting a standardized mucolytic preparation (Sputolysin, Calbiochiem, Sigma-Aldrich, Milan, Italy, Cat.#560000). After this addition, vortexing was performed for approximately 5 min at room temperature to uniformly mix the DTT and sample. The samples were then incubated in a thermostatically controlled water bath at 37 °C until the mucus component was completely liquefied. This last process took approximately 2 h on average. During this time, the sample was visually inspected every 10 min. The soluble phase of the sputum was obtained by centrifugation (13,000 rpm for 10 min at 4 °C), and the supernatant was stored at −20 °C until analysis. Sputum samples not treated with DTT were simply centrifuged at 10,000× *g* for 15 min at 4 °C, and the supernatants were immediately stored at −20 °C. To match the sevenfold dilution factor of the second protocol, samples from the first protocol were diluted accordingly prior to the ELISA.

ELISA assays for HMGB1 and D-dimer (Invitrogen, Thermo Fisher Scientific, Waltham, MA, USA) were performed on all samples (HMGB1: *n* = 9 for SE; *n* = 9 for SPE; *n* = 7 for SED. D-dimer: *n* = 8 for SE; *n* = 9 for SPE; *n* = 5 for SED). The human ELISA HMGB1 kit (Cat.#EEL047) has an assay range of 0–2000 ng/mL and a sensitivity of 0.01875 ng/mL. The human ELISA D-dimer kit (Cat.#EHDDIMER) has an assay range of 0.082–60 pg/mL and a sensitivity of 0.08 pg/mL.

### 2.2. Statistical Analysis

The Shapiro–Wilk test was performed to verify the normal distribution of data within each group before statistical analysis. Based on the non-normality of data, comparisons between groups were performed by Kruskal–Wallis test followed by Mann–Whitney test. The data are presented as medians, interquartile range, minimum and maximum values. A *p*-value < 0.05 has been considered statistically significant. Spearman rank correlation coefficients (r_s_) between parameters were computed. GraphPad Software (version 9.0, GraphPad Software) was used for the analysis.

## 3. Results

### 3.1. Clinical Characteristics of CF Patients

The selected patients (*n* = 13) were all homozygous or heterozygous with the *F508del* mutation, aged >18 years, and with a body mass index (BMI) indicating normal weight. Although the age at diagnosis was very young, Trikafta therapy was initiated at a median age of 23 years. The median forced expiratory volume in the first second (FEV_1_), a parameter of the respiratory function, was normal but ranged below normal (70%), indicating that some patients had poor respiratory function. All patients presented with bronchiectasis. The subjects had respiratory infections caused by bacteria typical of this disease, namely *Pseudomonas aeruginosa*, *Burkholderia cepacia*, and *Staphylococcus aureus*. Hematological parameters did not show significant alterations compared to normal values (Table 1).

### 3.2. HMGB1 and D-Dimer Levels in Sputum Samples

Although twenty sputum samples were submitted to the laboratory, seven of them were excluded because the salivary component was overwhelming compared to the very small mucosal component. The average starting volume of each sputum sample was approximately 500 μL. In our experimental procedure, some sputum samples were immediately centrifuged, obtaining supernatant and pellet. The supernatant was directly subjected to ELISA determination of HMGB1 and D-dimers; in the remainder of the Discussion, this type of sample will be referred to with the acronym “SE”. The pellet obtained after the aforementioned centrifugation was treated with DTT, as described in the Section 2, and then centrifuged to obtain a supernatant for ELISA determinations; in the remainder of the Discussion, this type of sample will be referred to by the acronym “SPE.” Other sputum aliquots were first treated with DTT, and then centrifuged, and the resulting supernatants were subjected to ELISA determinations; in the remainder of the Discussion, this type of sample will be referred to by the acronym “SED.”

**Figure 1:**
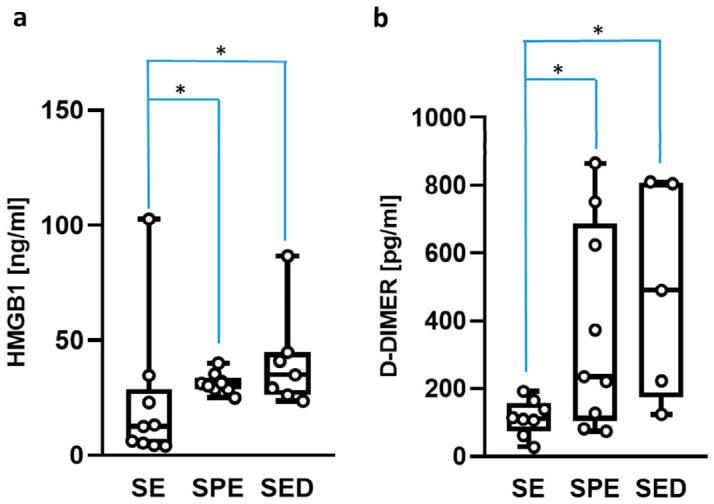
HMGB1 and D-dimer levels in sputum samples. Sputum was processed to obtain SE, SPE, and SED samples as detailed in the Materials and Methods, and assayed for HMGB1 (**a**) and D-dimer (**b**) levels by ELISA assays. The reported values were obtained by multiplying the raw ELISA data by a factor of seven. Values are shown as medians, interquartile range, minimum and maximum values. Mann–Whitney test: * *p* < 0.05.

Normality testing using the Shapiro–Wilk method revealed a non-normal distribution of the data (*p* < 0.05). Therefore, non-parametric tests were applied. Figure 1 reports HMGB1 and D-dimer levels as assessed on our samples by ELISA assay. Median HMGB1 levels were significantly lower in supernatants from centrifuged sputum before DTT treatment (SE) compared to both SPE supernatants and SED supernatants (Figure 1a). It can be highlighted, however, that SPE and SED samples were not different.

To note, several samples approached or exceeded the upper quantification limit for D-dimer levels. As in the case of HMGB1, the median detectable D-dimer levels were significantly lower in the supernatants from centrifuged sputum before DTT treatment (SE) compared to both SPE and SED supernatants. The difference between SPE vs. SED samples was not significant (Figure 1b).

The Kruskal–Wallis test gave an overall *p*-value of 0.0291 for HMGB1 and 0.0198 for D-dimer levels. The exact levels of each biomarker and the exact *p*-values, computed by Mann–Whitney test, are reported in Table 2.

**Figure 2:**
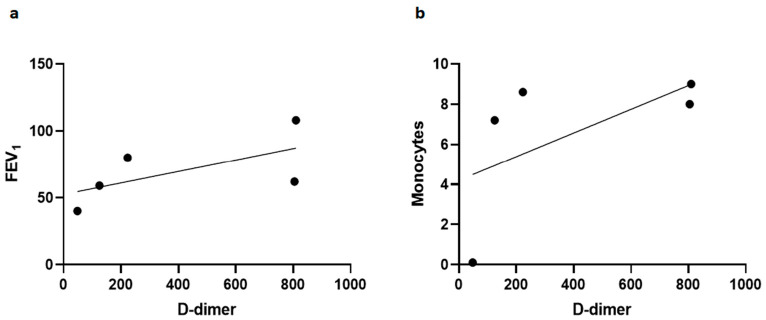
Correlation of D-dimer SED with FEV_1_ and monocytes. (**a**) Positive correlation between FEV_1_ and D-dimer levels (pg/mL). (**b**) Positive correlation between monocytes (% of total WBC) and D-dimer levels (pg/mL).

Next, we considered whether HMGB1 and D-dimer sputum levels correlated with clinical parameters (FEV_1_, BMI, CRP, WBC, and differential counts). While HMGB1 in all the three samples did not correlate with any parameter, D-dimer SED correlated positively with FEV_1_ and monocytes. In both cases, a strong trend toward a positive correlation was observed between the two parameters (r_s_ = 0.90), which, however, did not reach statistical significance (*p* = 0.08), likely due to the limited sample size. Figure 2 represents these correlations.

## 4. Discussion

The identification of biomarkers in cystic fibrosis could elucidate the pathophysiological stages of the disease, aiding in the diagnostic stratification of patients as well as in drug development [17] and therapeutic monitoring [18]. Few inflammatory markers have so far been identified, with NE, IL-8, IL-1β and TNF-α having the greatest responsiveness to therapeutic interventions, although endowed with variability [14]. More targeted approaches are needed for further identify biomarkers of inflammation and tissue repair in CF.

HMGB1 and D-dimers are of a certain relevance for CF, as both biomarkers reflect the chronic inflammatory state and pulmonary hypercoagulability characteristic of the pathology. HMGB1 is an alarmin released by damaged cells and neutrophils. It is fundamental to understanding the pathophysiological mechanisms of the disease, as it correlates directly with the extent of pulmonary damage [19]. D-dimers are by-products derived from the cleavage of cross-linked, insoluble fibrin molecules. An intricate relationship between inflammation, coagulation, and fibrinolysis exists [20]. Pro-inflammatory cytokines (such as IL-6, TNF-α, and IL-1β) stimulate the expression of tissue factor (TF) on monocytes and endothelial cells, initiating the extrinsic coagulation cascade. On the other hand, coagulation modulates inflammation. Activated coagulation factors, particularly thrombin, factor Xa, and the TF-factor VIIa complex, and components of the plasminogen–plasmin system act on protease-activated receptors (PARs) on inflammatory cells, boosting the production of pro-inflammatory cytokines [20,21,22]. Moreover, while early inflammation may enhance fibrinolytic activity, sustained inflammation increases plasminogen activator inhibitor-1 (PAI-1) levels, which effectively stops fibrin removal and promotes persistent, pathological clot formation [23]. However, whether these interactions between coagulation and inflammation that have been quite firmly established in systemic inflammation also operate and influence localized inflammatory processes such as in CF remains to be established [24].

Here we used the spontaneous sputum as a non-invasive matrix for biomarker discovery in CF, serving for elucidating whether a reducing agent can permit a sensitive detection of HMGB1 and D-dimer levels. Prior to biomarker analysis, sputum must undergo solubilization using mucolytic agents (such as dornase alfa), reducing agents [DTT and dithioerythritol (DTE)] or mechanical dissociation [14]. DTT (commonly used at 0.1% to 10 mM) disrupts the gel-like structure of mucus (e.g., MUC5AC, MUC5B), transforming it into a fluid state suitable for analysis. It effectively disperses cell clusters trapped in mucus, facilitating accurate cell counting, differential cell analysis, gene expression and flow cytometry [25,26].

Sputum samples were prepared for ELISA analysis by two protocols: direct solubilization with DTT (SED) or first separation of the solid fraction from the soluble one. In the second protocol, the former insoluble fraction was further processed by DTT (SPE) while the last soluble fraction was assayed as such (SE). For both HMGB1 and D-dimers, treatment with DTT resulted in significantly higher measured levels, indicating that these biomarkers are not freely available in untreated sputum. However, given that even highly viscous CF sputum is characterized by a typical mesh size on the order of hundreds of nanometers [27], substantially larger than the hydrodynamic dimensions of both HMGB1 and D-dimer, a purely steric trapping mechanism within the mucin network appears insufficient to fully explain this observation. A more plausible interpretation is that HMGB1 and D-dimers are retained within the mucus matrix primarily through non-specific interactions with mucins, including electrostatic and hydrophobic bonds, which significantly hinder their diffusion and accessibility. In this framework, the effect of DTT extends beyond a simple increase in mesh size: by reducing disulfide bonds, DTT alters the molecular organization of mucus, leading to a partial reorganization of the polymeric network and a reduction in effective binding sites for embedded proteins. Such behavior aligns with recent physical descriptions of native mucus as a heterogeneous material whose structure arises from arrested phase separation, and where reduction in disulfide bonds induces not only fluidization but also the emergence of macroheterogeneities and network reorganization. These structural transitions significantly impact molecular sequestration and transport within mucus matrices, providing a mechanistic basis for the enhanced detectability of biomarkers following DTT treatment [28,29].

Interestingly, SPE and SED samples were not different in biomarker levels, although the direct solubilization (SED) obtained slightly higher levels than SPE, suggesting that an aliquot of both markers remained insoluble. To strengthen the procedure that allowed us to obtain meaningful levels of these biomarkers, D-dimer levels in SED strongly correlated positively with FEV_1_ and monocyte counts. Although not statistically significant, the high correlation coefficient (r_s_ = 0.90) suggests a strong biological association that warrants further investigation. Future studies with a larger sample size are needed to confirm these findings and achieve adequate statistical power. Nevertheless, this might mean that the D-dimers can be accounted as a marker of increased respiratory function; i.e., the more fibrinolysis, the better the lung function. These results should be corroborated by measures involving fibrinolytic molecules, such as tissue-type plasminogen activator and plasminogen/plasmin. Monocyte-derived macrophages are actuated in the resolution of inflammation [30]. Our results indicate that monocytes can collaborate in the resolution of inflammation by degrading the fibrin clot. Indeed, monocytes and macrophages plays a key role in fibrinolysis by assembling components of the fibrinolytic system on their surface and also by internalization of fibrin and its degradation [31]. The fact that HMGB1 was not correlated with clinical parameters reflects the divergent results obtained from other studies. Indeed, Tiringer et al. [5] showed that HMGB1 levels in BALF were significantly higher in non-CF controls with recurrent infections than in stable CF patients, while Rowe et al. [8] showed that sputum HMGB1 was higher in CF patients in acute exacerbations as compared with healthy controls. Our patients are in stable conditions due to Trikafta treatment, and thus overall these data indicate that HMGB1 would be worthwhile to measure in CF patients in acute exacerbation.

The limit of this work is the small number of CF patients, although highly homogeneous from the genetic and clinical viewpoints, which should be improved in subsequent studies, above all for considerations about the clinical usefulness of the biomarkers considered in this study. Another issue arose out of the observation that several samples approached or exceeded the upper quantification limit for D-dimer levels, suggesting that D-dimer concentrations in CF sputum may be higher than anticipated and warrant further investigation using assays specifically validated for this biological matrix.

Our findings support the potential utility of sputum-based biomarker analysis as a non-invasive approach for disease phenotyping, monitoring disease progression, and evaluating treatment efficacy. Future studies involving larger and longitudinal cohorts are warranted to validate these observations and to establish the clinical utility of HMGB1 and D-dimer as biomarkers in routine CF care.

## 5. Conclusions

The direct solubilization of sputum samples represents a promising strategy for the assessment of HMGB1 and D-dimer levels in patients with cystic fibrosis. The ability to quantify biomarkers reflecting cellular damage, inflammation and coagulation/fibrinolitic activation directly within the respiratory tract offers important insights into complex mechanisms underlying CF lung disease. In this regard, we could measure HMGB1 and D-dimer levels in CF sputum by a simple protocol involving DTT, with the meaningful results that D-dimers, an underappreciated marker in CF lung disease, were also measurable.

## Figures and Tables

**Table 1:** Caracteristics of CF patients.

	N.	13
Anthropometric Data		
	Sex (M/F)	7/6
	Age (years)	27 (23–45)
	BMI (kg/m^2^)	23.5 (20.1–24.3)
Genotype		
	*F508del*/*F508del* (*n*)	4
	*F508del*/other mutation (*n*)	9
Clinical Data		
	Age at diagnosis (years)	1 (0.3–5.8)
	Age of starting triple therapy (years)	23 (20.5–41.0)
	FEV_1_% predicted	69 (57–86)
	Bronchiectasis	13/13
Respiratory Infections		
	*Pseudomonas aeruginosa* (*n*)	4
	*Burkholderia cepacia* (*n*)	2
	*Staphylococcus aureus* (*n*)	2
	*MRSA* (*n*)	1
	*Escherichia coli* (*n*)	1
Haematological Parameters		
	Reactive Protein C (mg/L)	0.38 (0.04–0.67)
	White Blood Cells (*n*/mm^3^)	5.5 (4.8–7.7)
	Neutrophils (%)	56.5 (45.2–65.1)
	Lymhocytes (%)	32.4 (22.5–39.7)
	Monocytes (%)	7.3 (6.5–9.6)
	Basophils (%)	0.55 (0.22–0.85)
	Eosinophils (%)	2.9 (1.1–4.2)

MRSA: Multidrug Resistance *Staphylococcus aureus*. Data are reported as medians (interquartile range).

**Table 2:** Levels of HMGB1 (ng/mL) and D-dimer (pg/mL) in CF sputum samples.

	Median	Interquartile Range	Min.–Max. Values	*p*-Value
HMGB1				
SE	12.53	4.829–28.78	4.088–102.7	–
SPE	31.16	28.76–33.70	24.89–40.80	SE vs. SPE: 0.0315
SED	34.91	26.25–44.86	23.58–86.64	SE vs. SED: 0.0229
D-dimer				
SE	112.0	73.09–158.0	27.35–192.3	–
SPE	235.3	105.1–687.5	74.76–865.0	SE vs. SPE: 0.0464
SED	490.5	173.9–807.1	124.9–809.6	SE vs. SED: 0.0109

## Data Availability

The raw data supporting the conclusions of this article will be made available by the authors on reasonable request.
